# Therapeutic effect of a temporary transpyloric stent in refractory post-surgical gastroparesis: a case report

**DOI:** 10.1186/s12893-019-0490-z

**Published:** 2019-02-27

**Authors:** Guo-Gang Liang, Qing-Kai Zhang, Gui-Xin Zhang, Mu-Cang Liu

**Affiliations:** Department of General Surgery, the First Affiliated Hospital of Dalian Medical University, Dalian, 116011 Liaoning Province China

**Keywords:** Case report, Post-surgical gastroparesis, Delayed gastric emptying, Self-expanding metallic stent, Temporary transpyloric stent

## Abstract

**Background:**

Gastroparesis is a syndrome characterized by delayed gastric emptying with associated symptoms. It was reported that the symptoms of diabetic gastroparesis had been greatly improved by transpyloric stent placement. However, the use of stents in benign conditions is considered to be contraindicated because of the increasing risk of complications, such as stent migration, reflux, perforation, bleeding, and, most importantly, new strictures caused by stent-induced tissue hyperplasia. While temporary placement of a self-expanding metallic stent (SEMC) can drastically reduce the risk of complications, few reports are available on the treatment of refractory PSG by temporary transpyloric stent. Does it have a long-term clinical effect after the stent being retrieved?

**Case presentation:**

After accepting partial resection of the lesser curvature in another hospital, a patient developed refractory gastroparesis. The symptoms hadn’t been improved after long-term drug therapy and balloon dilation therapy. Four months after surgery, a fully covered SEMC was placed by endoscopy in our hospital. Gastroparesis had been greatly improved. Two weeks later, the transpyloric stent was retrieved and the patient didn’t show recurrent symptoms. Follow-ups were arranged at 3 months, 6 months and 1 year respectively, and there was no evidence of recurrence was found.

**Conclusions:**

This case indicates that temporary transpyloric SEMC is a safe, effective and less invasive alternative for post-surgical gastroparesis patients.

## Background

Gastroparesis is a syndrome characterized by delayed gastric emptying with associated symptoms [1]. Medical treatment includes the use of prokinetic agents and anti-emetic agents. Endoscopic and surgical methods are used to treat refractory cases [1, 2]. Intrapyloric botulinum injection, pyloroplasty, partial gastrectomy, and most recently endoscopic techniques such as gastric peroral endoscopic myotomy are performed to relieve symptoms [3–6].

The most common etiologies of gastroparesis are idiopathic, diabetic, or post-surgical [7]. Post-surgical gastroparesis (PSG) is recognized as a consequence of vagal nerve injury following upper abdominal surgery. Initial postoperative management of PSG should be conservative as many symptoms following abdominal surgery resolve with time. Persistent symptoms are difficult to handle [8]. It was reported that the symptoms of diabetic gastroparesis had been greatly improved by transpyloric stent placement [9, 10]. However, the use of stents in benign conditions is considered to be contraindicated because of the increasing risk of complications, such as stent migration, reflux, perforation, bleeding, and, most importantly, new strictures caused by stent-induced tissue hyperplasia [11, 12]. Temporary placement of a fully covered self-expanding metallic stent (SEMC) as therapy for refractory benign esophageal strictures is recommended. The stent will be retrieved later and can drastically reduce the risk of complications [13]. However, few reports are available on the treatment of refractory PSG by temporary transpyloric stent. In this case report, the use of temporary transpyloric stent was described.

## Case presentation

**Fig. 1:**
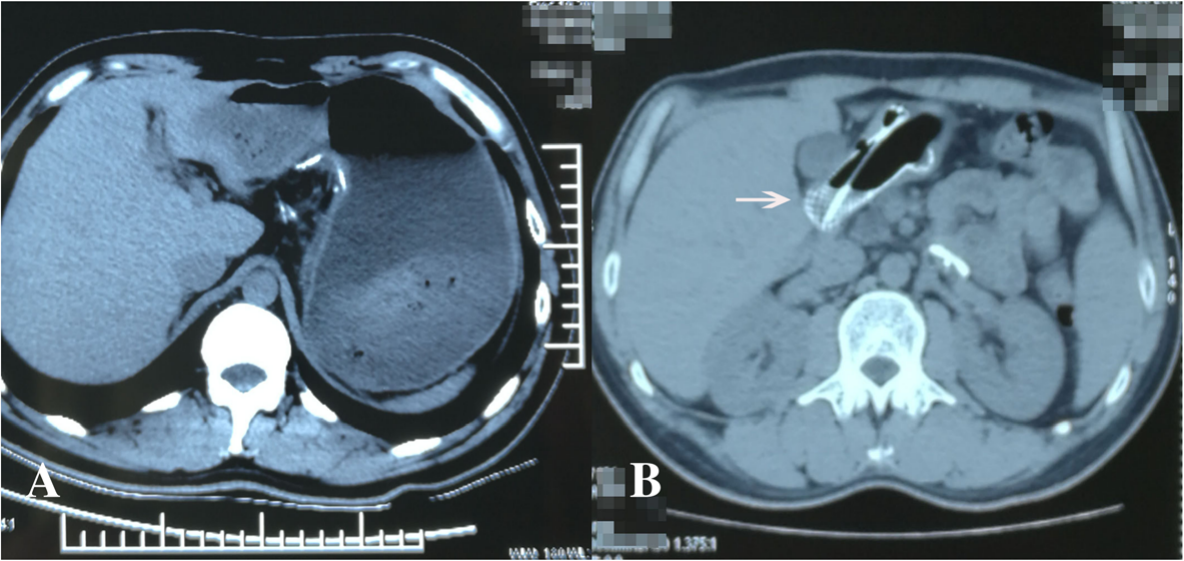
CT of the abdomen. **a** Gastric dilatation 12 h after a meal on the 3rd postoperative day. **b** Placement of a SEMC 4 months after surgery (→: SEMC)

**Fig. 2:**
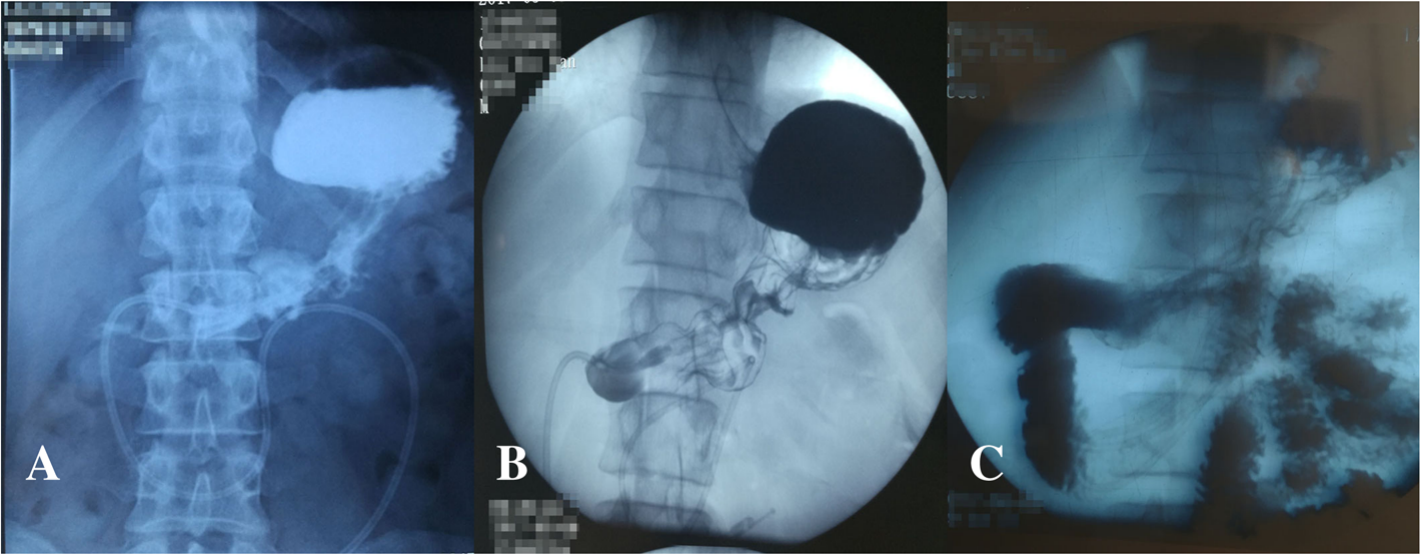
Upper gastrointestinal contrast study after surgery. **a** gastric peristalsis became weak and didn’t observe gastric emptying on the 16th postoperative day. **b** gastric peristalsis became weak and didn’t observe gastric emptying 4 months after surgery. **c** gastric emptying became good after SEMC placement

A 35-year-old patient was treated by pylorus-preserving gastrectomy for a gastric tumor at another hospital. The tumor was confirmed as heterotopic pancreas based on pathological findings. On the third post-operative day, the patient had epigastric distention and vomited several times after a meal, without abdominal tenderness and fever. A CT scan of the abdomen was performed and revealed massive gastric dilatation (Fig. 1a). The patient received gastric decompression and parenteral nutrition for 10 days, but had still epigastric distention and vomited after a meal. As a result, gastroparesis was diagnosed. On the 15th post-operative day, gastric decompression and jejunal enteral feeding were performed by using a Freka Trelumina (Zhejiang, China). The result of an upper gastrointestinal contrast study displayed that gastric peristalsis became weak and didn’t observe gastric emptying (Fig. 2a). Metoclopramide, erythromycin, and anti-emetic agents were administered, but no improvement in gastroparesis was observed. The patient underwent balloon dilation on the 40th post-operative day, but did not achieve the desired results. Several professional consultation had been adopted, and most of doctors thought the gastroparesis would be improved with time. But, gastroparesis wasn’t improved and the patient had some difficulty in oral nutrition in four months. Thus, he was transferred to our hospital for further treatment.

**Fig. 3:**
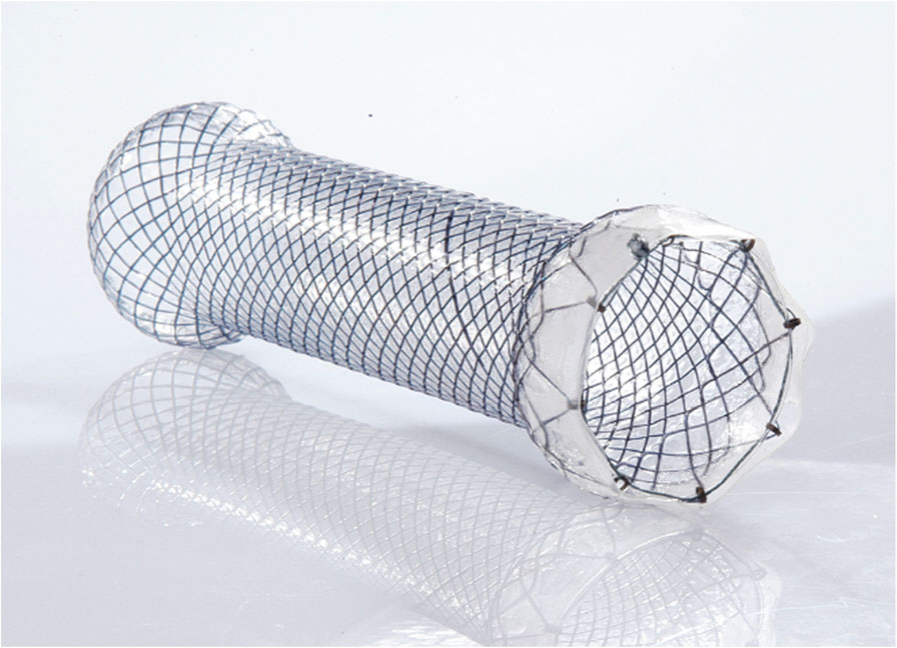
Fully covered self-expanding metallic stent

He suffered from severe epigastric distention even after drinking some water. On examination, the patient was very anxious. An upper gastrointestinal contrast study was performed. The result displayed that gastric peristalsis became weak and didn’t observe gastric emptying (Fig. 2b). Gastric endoscopy revealed that the pylorus kept continuous contraction. As a result, refractory gastroparesis was diagnosed. A fully covered SEMC (60 × 22 mm, MICRO∙TECH, Nanjing China) was placed by endoscopy and fluoroscopic guidance (Fig. 3, Fig. 1b). The stent was mounted in a compressed state on the guiding tube by the introducer sheath. It could expand to its expected and maximal diameter at body temperature. Meanwhile, it could shrink and become soft at cold temperatures. An upper gastrointestinal contrast study displayed that gastric emptying became good after SEMC placement (Fig. 2c). On the first day after stent placement, gastric decompression juice was significantly reduced. On the second day, gastric decompression was stopped and liquid food was allowed without epigastric distention. On the fifth day, semiliquid food was allowed. Two weeks later, the transpyloric stent was retrieved. First, 500 ml ice-cold water was injected. Then, the stent was grasped and pulled out. The patient did not experience recurrent symptoms. After three days, the patient was discharged from our hospital. Follow-ups were arranged at 3 months, 6 months and 1 year respectively, and there was no evidence of recurrence was found.

## Discussion and Conclusion

PSG is recognized as a consequence of vagal nerve injury following upper abdominal surgery. It has been well documented by vagotomy for peptic ulcer. In this case, the patient developed refractory gastroparesis after partial resection of the lesser curvature. Vagal nerve injury may be involved in the pathogenesis of gastroparesis [8, 14, 15]. During pylorus preserving gastrectomy, if vagal nerve injury is unavoidable, additional pyloroplasty may be a good choice, in order to reduce the incidence of PSG [16, 17]. However, pyloroplasty is not the first choice for PSG due to the trauma of a second operation.

Gastroparesis is characterized by physiologic disturbances in antral hypomotility, increased gastric outlet resistance, and pyloric dysfunction without evidence of obstruction [18, 19]. In this case, upper gastrointestinal contrast study displayed gastric peristalsis became weak and didn’t observe gastric emptying 4 months after surgery. Gastric endoscopy revealed that the pylorus kept continuous contraction. This indicated that pyloric spasms may play a key role in refractory PSG.

The initial management of PSG should be conservative as many symptoms following abdominal surgery resolve with time. This occurs possibly because the enteric nervous system is able to adapt to the loss of vagal input or vagal reinnervation occurs. Persistent symptoms are difficult to handle and require a multidisciplinary team approach [8]. Pyloric sphincter therapies including botulinum toxin, balloon dilation, transpyloric stent placement, endoscopic pyloromyotomy and surgical pyloroplasty have been developed [3–6, 20, 21]. Temporary placement of a fully covered SEMC may be a good choice for refractory PSG. The body of the stent is covered with polyethylene which can prevent the tissue from growing into it. Thus it can be retrieved easily. The stent will be retrieved later and can drastically reduce the risk of complications [13, 22, 23]. However, few reports are available. Most worrying is that whether it will have a long-term clinical effect after the stent being retrieved. In this case, the patient’s symptoms was improved apparently after the SEMC being placed. No evidence of recurrence was found after the stent being retrieved. It indicates that temporary placement of a fully covered SEMC has a long-term clinical effect on refractory gastroparesis. As for the possible mechanism, we consider that it is attributed to slow tearing of the pyloric muscularis. After SEMC placement, at body temperature, the stent gradually expand to the expected diameter within 24 h, and the pylorus muscularis is torn slowly and regularly with relatively few scars during stent expansion. Therefore, even when the stent is retrieved, the incidence of pyloric spasms and stenosis is very low [23]. Reports about the time of stent being retained in the body are inconsistent. It was reported that fully covered self-expanding metallic stents for achalasia was retrieved 3–7 days after stent placement and had good therapeutic effect [23]. In our report, the time was two weeks and the results proved that two weeks is good choice. However, the best choice about time need more data.

This case indicates that pyloric spasms may play a key role in refractory PSG; as a less invasive treatment, temporary transpyloric SEMC is a safe and effective alternative for refractory PSG. However, data are limited and more studies are under way.

## Abbreviations

CT: Computed tomography
PSG: Post-surgical gastroparesis
SEMC: Self-expanding metallic stent

## References

[1] Camilleri M, Parkman HP, Shafi MA, Abell TL, Gerson L (2013). Clinical guideline: management of gastroparesis. Am J Gastroenterol.

[2] Hibbard ML, Dunst CM, Swanström LL (2011). Laparoscopic and endoscopic pyloroplasty for gastroparesis results in sustained symptom improvement. J Gastrointest Surg.

[3] Lee JH, Kim CG, Kim YW, Choi IJ, Lee JY, Cho SJ, Kim YI, Eom BW, Yoon HM, Ryu KW (2017). Botulinum toxin injection for the treatment of delayed gastric emptying following pylorus-preserving gastrectomy: an initial experience. J Gastric Cancer.

[4] Bae JS, Kim SH, Shin CI, Joo I, Yoon JH, Lee HJ, Yang HK, Baek JH, Kim TH, Han JK, Choi BI. Efficacy of gastric balloon dilatation and/or retrievable stent insertion for pyloric spasms after pylorus-preserving gastrectomy: retrospective analysis. PLoS One. 2015. 10.1371/journal.pone.0144470. 10.1371/journal.pone.0144470 PMC4675538 26657405

[5] Malik Z, Kataria R, Modayil R, Ehrlich AC, Schey R, Parkman HP, Stavropoulos SN. Gastric per Oral endoscopic Myotomy (G-POEM) for the treatment of refractory gastroparesis: early experience. Dig Dis Sci. 2018; 10.1007/s10620-018-4976-9. 10.1007/s10620-018-4976-9 29468376

[6] Kahaleh M, Gonzalez JM, Xu MM, Andalib I, Gaidhane M, Tyberg A, Saumoy M, Baptista Marchena AJ, Barthet M. Gastric peroral endoscopic myotomy for the treatment of refractory gastroparesis: a multicenter international experience. Endosc. 2018. 10.1055/a-0596-7199. 10.1055/a-0596-7199 29649841

[7] Navas CM, Patel NK, Lacy BE (2017). Gastroparesis: medical and therapeutic advances. Dig Dis Sci.

[8] Shafi MA, Pasricha PJ (2007). Post-surgical and obstructive gastroparesis. Curr Gastroenterol Rep.

[9] Clarke JO, Sharaiha RZ, Kord Valeshabad A, Lee LA, Kalloo AN, Khashab MA (2013). Through-the-scope transpyloric stent placement improves symptoms and gastric emptying in patients with gastroparesis. Endosc..

[10] Khashab MA, Besharati S, Ngamruengphong S, Kumbhari V, El Zein M, Stein EM, Tieu A, Mullin GE, Dhalla S, Nandwani MC, Singh V, Canto MI, Kalloo AN, Clarke JO (2015). Refractory gastroparesis can be successfully managed with endoscopic transpyloric stent placement and fixation (with video). Gastrointest Endosc.

[11] Radecke K, Gerken G, Treichel U (2005). Impact of a self-expanding, plastic esophageal stent on various esophageal stenoses, fistulas, and leakages: a single-center experience in 39 patients. Gastrointest Endosc.

[12] Tomita M, Saito S, Makimoto S, Yoshida S, Isayama H, Yamada T, Matsuzawa T, Enomoto T, Kyo R, Kuwai T, Hirata N, Shimada M, Hirakawa T, Koizumi K, Saida Y. Self-expandable metallic stenting as a bridge to surgery for malignant colorectal obstruction: pooled analysis of 426 patients from two prospective multicenter series. Surg Endosc. 2018. 10.1007/s00464-018-6324-8. 10.1007/s00464-018-6324-8 PMC6342866 30006840

[13] Spaander MC, Baron TH, Siersema PD, Fuccio L, Schumacher B, Escorsell À, Garcia-Pagán JC, Dumonceau JM, Conio M, de Ceglie A, Skowronek J, Nordsmark M, Seufferlein T, Van Gossum A, Hassan C, Repici A, Bruno MJ (2016). Esophageal stenting for benign and malignant disease: European Society of Gastrointestinal Endoscopy (ESGE) clinical guideline. Endosc..

[14] Dong K, Yu XJ, Li B, Wen EG, Xiong W, Guan QL (2006). Advances in mechanisms of postsurgical gastroparesis syndrome and its diagnosis and treatment. Chin J Dig Dis.

[15] Camilleri M (2016). Functional dyspepsia and gastroparesis. Dig Dis.

[16] Mancini SA, Angelo JL, Peckler Z, Philp FH, Farah KF (2015). Pyloroplasty for refractory gastroparesis. Am Surg.

[17] Shada AL, Dunst CM, Pescarus R, Speer EA, Cassera M, Reavis KM, Swanstrom LL (2016). Laparoscopic pyloroplasty is a safe and effective first-line surgical therapy for refractory gastroparesis. Surg Endosc.

[18] Parkman HP, Hasler WL, Fisher RS (2004). American Gastroenterological Association. American Gastroenterological Association technical review on the diagnosis and treatment of gastroparesis. Gastroenterol..

[19] Friedenberg FK, Parkman HP (2007). Advances in the management of gastroparesis. Curr Treat Options Gastroenterol..

[20] Clarke JO, Snape WJ (2015). Pyloric sphincter therapy: botulinum toxin, stents, and pyloromyotomy. Gastroenterol Clin North Am.

[21] Ahuja NK, Clarke JO (2017). Pyloric therapies for gastroparesis. Curr Treat Options Gastroenterol.

[22] Coppola F, Gaia S, Rolle E, Recchia S (2014). Temporary endoscopic metallic stent for idiopathic esophageal achalasia. Surg Innov.

[23] Zhao J-G, Li Y-D, Cheng Y-S, Li M-H, Chen N-W, Chen W-X, Shang K-Z (2009). Long-term safety and outcome of a temporary self-expanding metallic stent for achalasia: a prospective study with a 13-year single-center experience. Eur Radiol.

